# SIK1 Regulates CRTC2-Mediated Gluconeogenesis Signaling Pathway in Human and Mouse Liver Cells

**DOI:** 10.3389/fendo.2020.00580

**Published:** 2020-09-02

**Authors:** Chang Wang, Daofei Song, Jiahui Fu, Xiuying Wen

**Affiliations:** ^1^Department of Endocrinology, Liyuan Hospital, Tongji Medical College, Huazhong University of Science and Technology, Wuhan, China; ^2^Department of Endocrinology, Hubei Provincial Hospital of Integrated Chinese and Western Medicine, Wuhan, China

**Keywords:** SIK1, CRTC2, hepatic gluconeogenesis, nuclear translocation, HepG2, mouse primary hepatocytes

## Abstract

The regulation of hepatic gluconeogenesis is of great significance to improve insulin resistance and benefit diabetes therapy. cAMP-Regulated Transcriptional Co-activator 2 (CRTC2) plays a key role in regulating hepatic gluconeogenesis through controlling the expression of gluconeogenic rate-limiting enzymes such as glucose-6-phosphatase (G6Pase) and phosphoenolpyruvate carboxykinase (PEPCK). Recently, salt-inducible kinase 1 (SIK1) has been identified to play an important role in glucose metabolism disorders, but whether and how SIK1 regulates the CTRC2 signaling in liver cells under high glucose conditions has rarely been intensively elucidated. Here, we show that high glucose stimulation resulted in time-dependent down-regulated expression of SIK1, phosphorylated SIK1 at T182 site, and phosphorylated CRTC2 at S171 site, as well as upregulated expression of total CRTC2 and its downstream targets G6Pase and PEPCK in the human liver cell line HepG2. The nuclear expression levels of SIK1 and CRTC2 were time-dependently upregulated upon high glucose challenge, which was accompanied by enhanced cytoplasm-to-nucleus translocation of SIK1. Manipulation of SIK1 activity using plasmid-mediated SIK1 over-expression and the use of the SIKs inhibitor HG-9-91-01 confirmed that SIK1 regulated the CRTC2 signaling pathway in HepG2 cells. Furthermore, in mouse primary hepatocytes, high glucose exposure down-regulated SIK1 expression, and promoted SIK1 nuclear accumulation. While HG-9-91-01 treatment suppressed SIK1 expression and released the inhibitory effects of SIK1 on the expressions of key molecules involved in the CRTC2 signaling pathway, additional ectopic expression of SIK1 using adenovirus infection reversed the impacts of HG-9-91-01 on the expressions of these molecules in mouse hepatocytes. Therefore, SIK1 regulates CRTC2-mediated gluconeogenesis signaling pathway under both physiological and high glucose-induced pathological conditions. The modulation of the SIK1-CRTC2 signaling axis could provide an attractive means for treating diabetes.

## Introduction

Gluconeogenesis is the process of glucose synthesis using non-sugar precursors and is critical in maintaining stable human blood glucose levels in humans. An abnormal increase of gluconeogenesis in the liver significantly promotes diabetic hyperglycemia (1). Glucose biosynthesis in the liver is regulated by multiple transcription factors such as the cAMP-response element-binding protein (CREB) (2), which modulates two rate-limiting enzymes in hepatic gluconeogenesis, including glucose-6-phosphatase (G6Pase) and phosphoenolpyruvate carboxykinase (PEPCK) (3). Excessive liver glucose output represents a major inducer of hyperglycemia in type 2 diabetes mellitus (T2DM), and its inhibition can reduce blood glucose levels in patients with T2DM (4, 5). In the fasting state, the amount of glucose output by the liver due to gluconeogenesis accounts for about 65% of the total blood glucose levels (6). Therefore, the regulation of gluconeogenesis is of great significance to stabilize blood glucose, improve insulin resistance, and even manage diabetes mellitus and other disorders of glucose metabolism.

Previous studies showed that the signaling pathways mediated by SIK1 play critical functions in glucose metabolism disorders (7, 8). SIK1 belongs to the serine-threonine kinase family, and also is an AMPK-related protein kinase (8). SIK1 is found in the nuclear and cytoplasmic compartments of resting mouse adrenocortical tumor cells, and adrenocorticotropic hormone (ATCH) stimulation induces the auto-phosphorylation at the Ser577 phosphorylation site, which causes nuclear export, and reduces the inhibitory effects of SIK1 on cAMP-responsive element (CRE)-associated gene expression (9). In addition, liver kinase B1 (LKB1) can upregulate SIK1 activity by phosphorylation at the Thr182 site in the SIK1's kinase domain (10, 11). Phosphorylation at the above SIK1 sites is closely related to its activity, intracellular distribution, and function (10, 11). In human tissues, SIK1 is mainly expressed in the skeletal muscle, brain, adrenal gland, testis, adipose tissue, liver and myocardium, and contributes to the modulation of water-electrolyte, carbohydrate and lipid metabolic pathways, cell proliferation, biological clock, and other physiological functions (12–15).

Animal experiments also showed that SIK1 affects vascular remodeling and blood pressure regulation (16). An early study found that in rat hepatic cells, over-expression of SIK1 increases the phosphorylation level of cAMP-regulated transcriptional co-activators (CRTC2) at the Ser171 site, which causes CRTC2 to leave the nucleus and suppress its binding to CREB, thereby reducing CRTC2's ability to upregulate downstream genes involved in gluconeogenesis (3), including PEPCK and G6Pase. Previously, we found that the high-glucose culture of rat mesangial cell lines could relieve the inhibitory effect of SIK1 on type I activin receptor-like kinase 5 (ALK5) signaling, leading to the proliferation of mesangial cells and extracellular matrix accumulation (17). In addition, when SIK1 is upregulated, genes involved in lipid deposition and synthesis are down-regulated in HepG2 cells, including sterol regulatory element-binding protein-1c (SREBP-1c), fatty acid synthase (FAS), and acetyl CoA carboxylase (ACC) in HepG2 cells are down-regulated (18). Therefore, the physiological and pathological functions of SIK1 in metabolic diseases deserve further investigation. Nevertheless, whether and how SIK1 regulates the CRTC2-mediated gluconeogenesis signaling pathway in liver cells has not been elucidated.

In this study, using the human liver HepG2 cell line and mouse primary hepatocytes as *in vitro* cell models, we examined the changes in SIK1 expression and phosphorylation, as well as the expression levels of key molecules involved in the CRTC2 signaling pathway, in response to high glucose exposure. Using a SIKs inhibitor and plasmid transfection- or adenovirus infection-mediated ectopic expression of SIK1 in liver cells, SIK1's impacts on the cellular location and expression levels of hepatic gluconeogenesis-associated key molecules were assessed.

## Materials and Methods

### Cells and Treatments

The HepG2 cell line was provided by the China Center for Type Culture Collection (China), and was maintained in Dulbecco's Modified Eagle's Medium (DMEM; Hyclone, USA) supplemented with a normal concentration of glucose (5.5 mmol/l D-glucose), 10% fetal bovine serum (FBS; Biological Industries, Israel), 100 μg/ml streptomycin, and 100 U/ml penicillin (Biosharp, China) at 37°C in a humidified atmosphere containing 5% CO_2_. At 70–80% confluency, the cells were cultured in medium without FBS for 12 h, and then treated with a high concentration of glucose (25 mmol/l D-glucose) for 6, 12, and 24 h, after incubation under normal glucose for 18, 12, and 0 h, respectively as previously described (total incubation of 24 h) (18). The SIKs inhibitor HG-9-91-01 was purchased from MedChemExpress (China).

### Cell Transfection

Full-length cDNA encoding the human SIK1 protein (GenBank-ID: BC038504) underwent amplification by reverse transcription-polymerase chain reaction (RT-PCR), with 5′-GAA CCG TCA GAT CCG CTA GCC GCC ACC ATG GTT ATC ATG TCG GAG TTC AG-3′ and 5′-TCA CCA TGG TGG CGA CCG GTG GCT GCA CCA GGA CAA ACG TGC CTA GG-3′ as forward and reverse primers, respectively. The SIK1 coding sequence was cloned into the mammalian expression vector pGV230 (Shanghai Gene Chemical Technology, China). HepG2 cells were transiently transfected with the human SIK1-overexpressing pGV230-SIK1 vector with Lipofectamine 2000 (Invitrogen, USA), according to the manufacturer's instructions. Briefly, HepG2 cells in 6-well plates were cultured until ~80% confluency. The cells were left untransfected or were transfected with the empty vector pGV230 or the pGV230-SIK1 plasmid. At 6 h after transfection, the culture medium was changed to regular DMEM with normal glucose levels. The cells were further cultured for 42 h and used for the experiments below.

### Isolation of Primary Mouse Hepatocytes

Specific pathogen-free (SPF) C57BL/6J mice (male, 6–8 weeks) provided by Beijing HFK Bioscience (China) were housed at the laboratory animal center of Tongji Medical College, Huazhong University of Science and Technology (Wuhan, China) at 22 ± 1°C under a 12–12 h light/dark cycle, and with freely available rodent chow and water. Primary mouse hepatocytes were obtained as described in a previous report (19). All experiments involving animals were approved by the University Committee on the Use and Care of Animals (UCUCA) of the Research Ethics Committee of Tongji Medical College, Huazhong University of Science and Technology.

### Adenovirus Production and Infection of Mouse Primary Hepatocytes

The adenovirus was produced based on a previous report (20). Briefly, Ad-SIK1 and negative control adenoviruses with an enhanced green fluorescent protein (Ad-EGFP) were manufactured by GeneChem (China). The recombinant adenoviruses were obtained at a titer of 1 × 10^9^ plaque-forming units (PFU)/ml. Primary mouse hepatocytes underwent culture (6-well plates) for 24 h and infection with the indicated adenoviruses at a multiplicity of infection (MOI) of 16. At 24 h after viral infection, the medium was changed to fresh DMEM. The infection ratio was validated to be over 90% by observing the EGFP signals under a fluorescence microscope at 48 h after infection.

### Quantitative Reverse Transcription-Polymerase Chain Reaction (qRT-PCR)

The total RNA from HepG2 cells and mouse primary hepatocytes was isolated using RNAiso Plus (Takara, Japan). Reverse transcription was carried out with RevertAid First Strand cDNA Synthesis Kit (Thermo Fisher Scientific, USA). The primers (Wuhan Qinke Innovation Biotechnology, China) are described in Table 1. The annealing temperature for SIK1 amplification was 52.5°C, while the internal control gene β-actin was amplified at 50°C. The PCR products were separated by agarose gel electrophoresis and visualized on a JS-680B-Imaging System (Shanghai Peiqing Science and Technology, China). SIK1 mRNA amounts in each sample were normalized to β-actin expression. Analysis was performed by densitometry with ImageJ (National Institutes of Health, USA).

**Table 1:** The sequences of primers used for PCR in this study.

**Species**	**Gene name**	**NCBI Acc#**	**Primer name**	**Primer sequence (5^**′**^-3^**′**^)**	**Product length**
Human	GAPDH	NM_002046	Forward	GGAAGCTTGTCATCAATGGAAATC	168
			Reverse	TGATGACCCTTTTGGCTCCC	
Human	SIK1	NM_173354.5	Forward	CGCCATGTATAGTCGTCTCCC	298
			Reverse	GCCTTCAGCCCTTGAGTCAGT	
Human	CRTC2	NM_181715.3	Forward	ATACACCCGCCACATTGACA	266
			Reverse	TTTCACCATCCAGAATACCCCC	
Human	PEPCK	NM_001018073.3	Forward	GTGGGGGATGATATTGCTTG	169
			Reverse	TGGTCTCAGCCACATTGGTA	
Human	G6pase	NM_000151.4	Forward	GGGTGTAGACCTCCTGTGGA	187
			Reverse	GAGCCACTTGCTGAGTTTCC	
Mouse	GAPDH	NM_008084.2	Forward	CCTCGTCCCGTAGACAAAATG	133
			Reverse	TGAGGTCAATGAAGGGGTCGT	
Mouse	G6PC	NM_008061.4	Forward	ACACCGACTACTACAGCAACAGC	208
			Reverse	AATCCCAACCACAAGATGACG	
Mouse	Pepck	NM_011044.3	Forward	GTGTTTACTGGGAAGGCATCG	223
			Reverse	ACACCTTCAGGTCTACGGCCA	
Mouse	Acc	NM_133360.2	Forward	GAGAACCCGAAACTCCCAGAAC	121
			Reverse	CTGCAGTTTGAGCCACAATAGAA	
Mouse	FAS	NM_007988.3	Forward	TGAATCAGCCCCACGCAGT	297
			Reverse	CCGAGTCAGTCTTGGAGGACAT	
Mouse	Sik1	NM_010831.3	Forward	CTACAACCACTTTGCCGCCAT	279
			Reverse	AGGGGGAATAATAAGGGCTGAAG	

qRT-PCR was carried out with an SYBR Green PCR kit (Toyobo, Japan) on an AB7500 RT-PCR (Applied Biosystems, USA), using the housekeeping gene GAPDH for normalization. The primers used for quantitating human and mouse genes by qRT-PCR are listed in Table 1.

### Immunoblotting

Total cell protein extraction was performed with the radioimmunoprecipitation assay (RIPA) lysis buffer (Beyotime Biotechnology, China) supplemented with phenylmethanesulfonyl fluoride (PMSF) and phosphatase inhibitors cocktail (Wuhan Servicebio technology, China). A nucleoprotein extraction kit (Hangzhou Fude Biological Technology, China) was used to isolate nucleoproteins, according to the manufacturer's instructions. Protein amounts were quantitated with a BCA protein assay kit (Hangzhou Fude Biological Technology, China). Equal amounts of proteins were resolved by 10% sodium dodecyl sulfate-polyacrylamide gel electrophoresis (SDS-PAGE) and electro-transferred onto nitrocellulose membranes. After blocking with 5% fat-free milk in Tris-buffered saline containing Tween-20 (TBS-T) for 2 h at ambient, overnight incubation was carried out with primary antibodies raised in rabbits against SIK1 (Proteintech Group, USA) (1:1000), SIK1 phospho-Thr-182 (Proteintech Group; 1:800), CRTC2 (Proteintech Group; 1:1000), CRTC2 phospho-Ser-171 (Proteintech Group; 1:800), G6Pase (Abcam, USA; 1:100), PEPCK (Cell Signaling; 1:500), mouse SREBP-1c (Proteintech Group; 1:500), mouse FAS (Proteintech Group; 1:500), mouse ACC (Proteintech Group; 1:500), TATA-binding protein (TBP) antibody (Proteintech Group; 1:1000), and β-actin (Cell Signaling; 1:1000) at 4°C, respectively. This was followed by incubation with goat anti-rabbit IgG (Proteintech Group; 1:5000) for 1 h at room temperature. Finally, band visualization was performed using an enhanced chemiluminescence (ECL) kit (EMD Millipore, USA). Immunoreactive bands were quantitated by densitometry with ImageJ and normalized to β-actin signals.

### Immunocytochemistry

HepG2 cells and mouse primary hepatocytes after the indicated treatments on glass coverslips underwent fixation with 4% formalin for 20 min, followed by immunocytochemistry for PEPCK. The samples were incubated with primary rabbit anti-human/mouse PEPCK antibodies (Cell Signaling; 1:50) at 4°C overnight. After washing with PBS containing 0.5% Tween-20 (PBS-T), secondary goat anti-rabbit antibodies (Proteintech Group; 1:500) were added at room temperature for 1 h. The diaminobenzidine substrate was used for development, and PEPCK protein expression was assessed microscopically.

### Immunofluorescence

The expression levels and cellular distributions of SIK1, CRTC2, and SREBP-1c in HepG2 cells and primary mouse hepatocytes were evaluated by immunofluorescence. Cells on glass coverslips were fixed with 4% formalin after the indicated treatments. Permeabilization was carried out with 0.5% Triton X-100, followed by blocking with PBS supplemented with 5% bovine serum albumin (BSA). Then, antibodies targeting SIK1 (Proteintech Group; 1:50), CRTC2 (Proteintech Group; 1:50), and SREBP-1c (Abcam; 1:50) in 5% BSA/PBS were added for incubation at 4°C overnight. Fluorescein isothiocyanate (FITC)- or tetramethylrhodamine (TRITC)-linked goat anti-rabbit antibodies (Wuhan Servicebio technology; 1:200) were used as secondary antibodies. Finally, 4',6-diamidino-2-phenylindole (DAPI) was used for counterstaining (15 min). A confocal microscope (Nikon Eclipse Ti, Japan) was used for imaging.

### Cell Viability Assay

The 3-(4,5-dimethylthiazol-2-yl)-2,5-diphenyltetrazolium bromide (MTT; Sigma, USA) assay was performed to assess the impact of high glucose on HepG2 cell viability. Cells in 96-well plates (5 × 10^3^ cells/well; *n* = 5) underwent culture in DMEM with normal and high concentrations of glucose, respectively, for 6, 12, or 24 h. This was followed by MTT (0.5 μg/mL) addition for incubation at 37°C for 4 h. Optical density at 492 nm was measured using a microplate reader. Cell viability percentage was assessed as [(A492_Sample_-A492_Blank_)/(A492_Control_-A492_Blank_)]×100. Cell viability levels of the samples in the experimental groups were normalized to that of control cells cultured in medium with normal glucose concentration.

### Statistical Analysis

Experimental data are shown as mean ± standard error of the mean (SEM) (x ± s) from at least three independent assays. GraphPad Prism 7.0 (GraphPad, USA) was used for data assessment by a one-way analysis of variance (ANOVA). *P* < 0.05 was considered statistically significant.

## Results

### High Glucose Decreases SIK1 Expression and Suppresses Nuclear Export in HepG2 Cells

**Figure 1:**
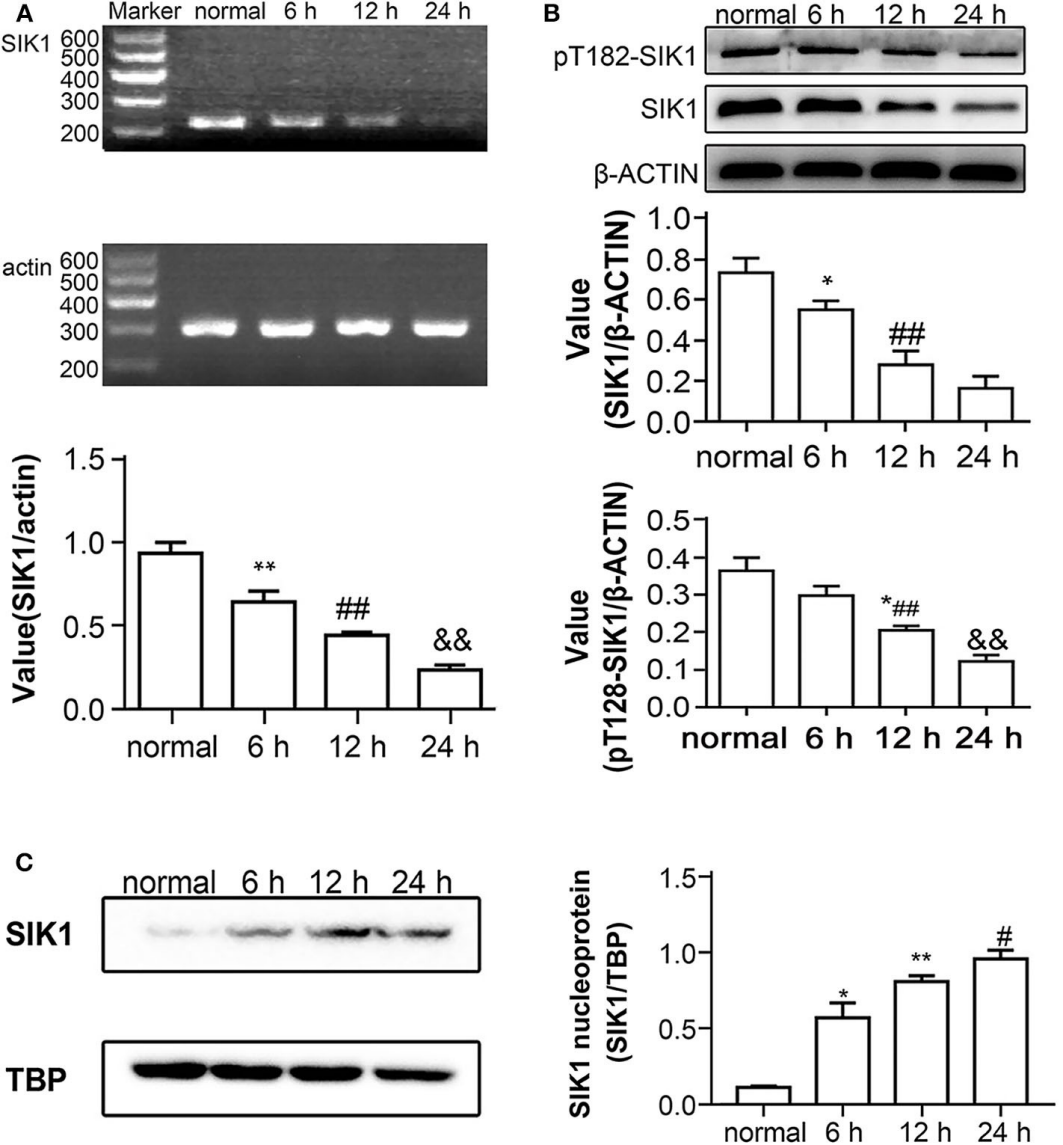
Effects of high glucose on SIK1 expression and nucleus/cytoplasmic location in HepG2 cells. **(A–C)** HepG2 cells were cultured in regular DMEM supplemented with a normal concentration of glucose (5.5 mmol/l D-glucose) or in DMEM supplemented with a high concentration of glucose (25 mmol/l D-glucose) for 6, 1,2, and 24 h. The expression levels of SIK1 mRNA were determined by semi-quantitative PCR with actin as the internal control gene **(A)**. The levels of total SIK1 protein and phosphorylated SIK1 protein at the T182 site were determined by immunoblotting **(B)**. The nuclear expression of SIK1 protein in the indicated HepG2 cells was determined by immunoblotting with TBP as the internal control protein for cell nuclear extracts **(C)**. Immunoblotting experiments were repeated at least three times, and one representative immunoblotting is shown for each experiment. *n* = 3 for bar graphs in **(A–C)**. *P < 0.05, **P < 0.01, compared with the normal glucose group; ^#^*P* < 0.05, ^##^*P* < 0.01, compared with the 6 h group; ^&^*P* < 0.05, ^&&^*P* < 0.01, compared with the 12 h group.

We first evaluated the impact of high glucose on SIK1 expression in HepG2 cells by qRT-PCR and immunoblotting. Compared with control cells cultured with a medium containing normal glucose amounts, the groups administered high glucose for 6, 12, and 24 h demonstrated decreased expression levels of SIK1, and this decrease appeared to have a time-dependent pattern (*P* < 0.05) (Figure 1A). Immunoblotting showed that the protein levels of total SIK1 and phosphorylated SIK1 at Thr182 were significantly decreased in HepG2 cells after high glucose stimulation (Figure 1B). Similarly, SIK1 amounts in the high glucose-treatment groups decreased gradually and in a time-dependent manner (*P* < 0.05) (Figure 1B).

SIK1 distribution in cells also reflects its functional activity (21). To confirm this phenomenon, we extracted the nucleoproteins from high-glucose-stimulated cells and performed immunoblotting assays. In comparison with the normal glucose group, the high glucose groups showed time-dependent increases in nuclear amounts of the SIK1 protein (*P* < 0.05) (Figure 1C). Collectively, the amounts of total SIK1 and phosphorylated SIK1 at Thr182 were decreased in HepG2 cells exposed to high glucose, which promoted the accumulation of total SIK1 in the nucleus, possibly through suppressing the nuclear export of SIK1.

### High Glucose Affects the CRTC2 Signaling Pathway in HepG2 Cells

**Figure 2:**
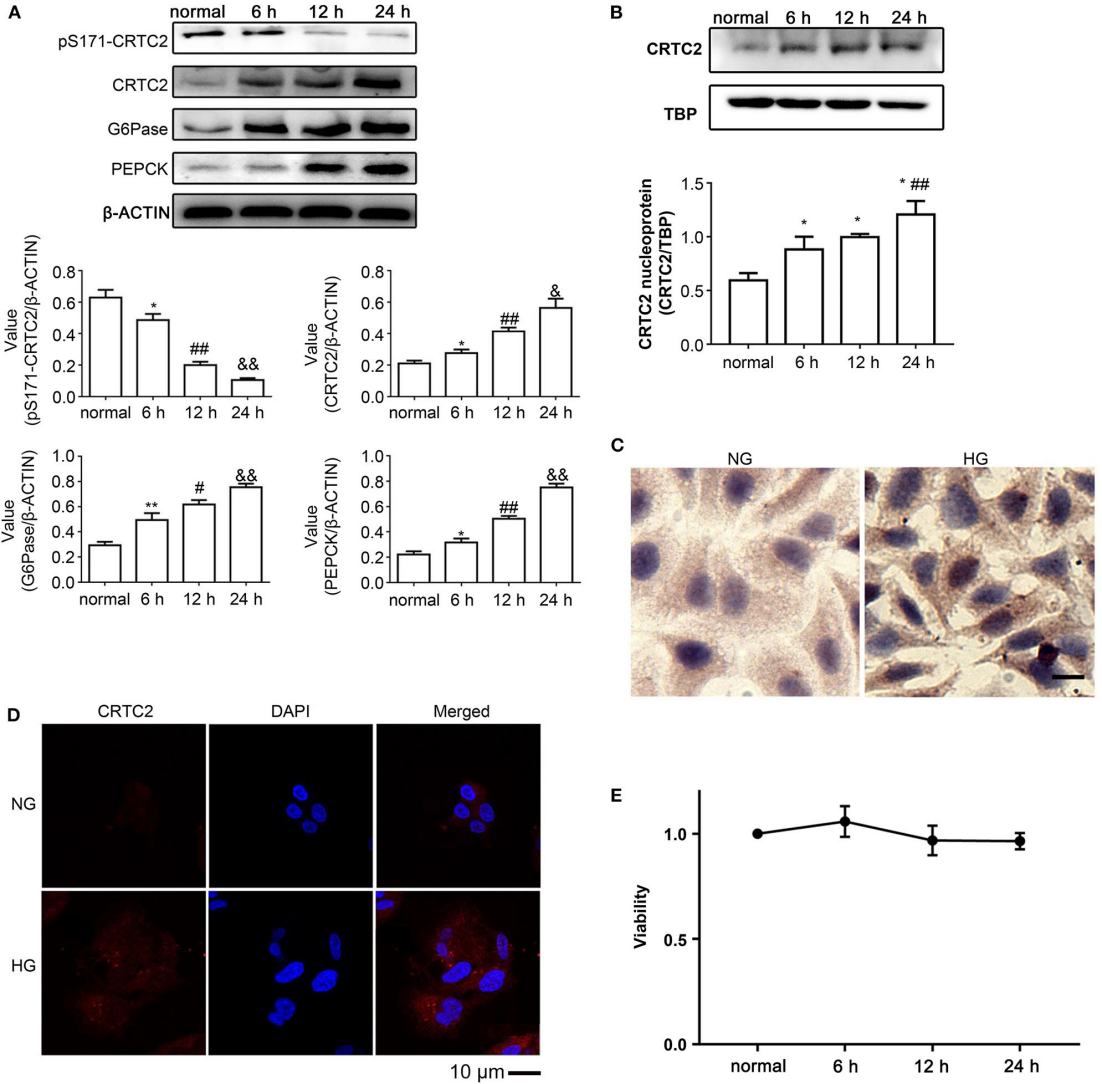
Effects of high glucose on the CRTC2 signaling pathway in HepG2 cells. **(A)** The protein levels of the CRTC2 signaling-associated molecules, including total CRTC2, phosphorylated CRTC2 at S171, G6Pase, and PEPCK were determined by immunoblotting assays. **(B)** The levels of nuclear CRTC2 in control HepG2 cells and HepG2 cells that underwent the indicated treatments were determined by immunoblotting with TBP as the internal control protein for cell nuclear extracts. **(C)** Representative images show the immunohistochemistry of PEPCK in HepG2 cells cultured under normal glucose and high glucose conditions for 24 h. **(D)** The distribution of CRTC2 in HepG2 cells after incubation in normal or high glucose medium for 24 h was evaluated by immunofluorescence. Scale bar, 10 μm. **(E)** The viability of HepG2 cells that underwent the indicated treatments were evaluated using the MTT method. Immunoblotting experiments were repeated at least three times, and one representative immunoblotting is shown for each experiment. *n* = 3 for graphs in **(A,B,E)**. *P < 0.05, **P < 0.01, compared with the normal glucose group; ^#^*P* < 0.05, ^##^*P* < 0.01, compared with the 6 h group; ^&^*P* < 0.05, ^&&^*P* < 0.01, compared with the 12 h group.

By phosphorylating CRTC2, SIK1 can lead to the nuclear export of CRTC2 and inhibit its binding to CREB, thereby down-regulating G6Pase and PEPCK (22). To examine the impacts of high glucose on CRTC2 signaling, the protein amounts of factors associated with this pathway were assessed by immunoblotting. In comparison with the normal glucose group, high-glucose treatment resulted in a time-dependent decrease in the phosphorylation levels of CRTC2 at the Ser171 site in HepG2 cells, while CRTC2 protein amounts were increased gradually upon high glucose challenge (Figure 2A). In addition, the protein expression levels of G6Pase and PEPCK were increased gradually in the high-glucose groups, and the above changes were also time-dependent (*P* < 0.05) (Figure 2A). These data indicated that SIK1's inhibitory effect on CRTC2 was weakened in a high glucose environment, which upregulated the rate-limiting gluconeogenic enzyme genes downstream of CRTC2 and promoted gluconeogenesis.

Next, we determined the nuclear expression of the CRTC2 protein. Immunoblotting showed that nuclear CRTC2 amounts after high-glucose treatment were increased in comparison with the normal glucose group (Figure 2B), corroborating the above findings suggesting that high glucose decreased the amounts of phosphorylated CRTC2 at Ser171. In agreement, immunocytochemistry also showed that in comparison with the normal glucose groups, the high glucose groups had markedly elevated expression of PEPCK (Figure 2C). Furthermore, immunofluorescence demonstrated that the cellular distribution of CRTC2 switched from a nucleus-dominant pattern to an even distribution between the cytoplasm and nucleus upon challenge with high glucose (Figure 2D). Exposure of HepG2 cells to the high glucose environment did not result in significantly decreased cell viability (Figure 2E).

### Overexpression or Inhibition of SIK1 Impacts the CRTC2 Signaling Pathway in HepG2 Cells

**Figure 3:**
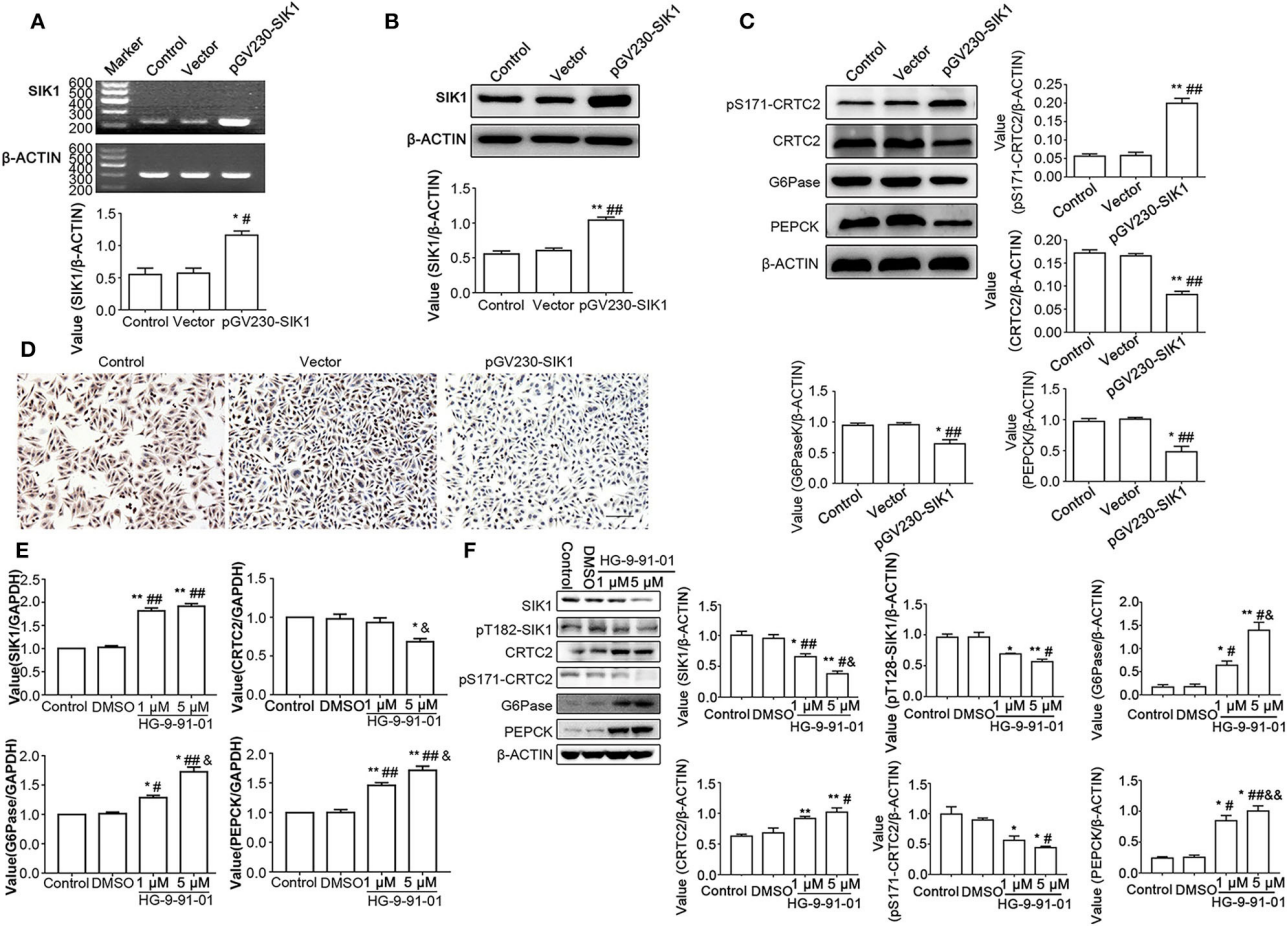
SIK1 overexpression or inhibition affects the CRTC2 signaling pathway in HepG2 cells. **(A–D)** HepG2 cells were mock-transfected (Control), or transfected with empty vector pGV230 (Vector), or transfected with SIK1-expressing vector (pGV230-SIK1), and cultured in regular medium with normal glucose concentration. At 48 h after transfection, the mRNA levels of SIK1 **(A)** and the protein levels of SIK1 **(B)** were determined by PCR and immunoblotting, respectively. The protein levels of the CRTC2 signaling-associated molecules were determined using immunoblotting **(C)**. The expression level of PEPCK in the indicated groups of HepG2 cells was evaluated by immunohistochemistry. Scale bar, 100 μm **(D)**. **(E,F)** HepG2 cells cultured in the regular medium with a normal glucose concentration were left untreated (Control), or treated with vehicle (DMSO), or treated with 1 or 5 μM SIKs inhibitor HG-9-91-01 for 48 h. The mRNA levels of SIK1, CRTC2, G6Pase, and PEPCP were determined by qPCR **(E)**. The protein levels of total SIK1, phosphorylated SIK1 (pT182-SIK1), total CRTC2, phosphorylated CRTC2 (pS171-CRTC2), G6Pase, and PEPCK were determined by immunoblotting **(F)**. Immunoblotting experiments were repeated at least three times, and one representative blotting result is shown for each experiment. *n* = 3 for graphs in **(A–C,E,F)**. *P < 0.05, **P < 0.01, compared with the Control group; ^#^*P* < 0.05, ^##^*P* < 0.01, compared with the Vector/DMSO group; ^&^*P* < 0.05, ^&&^*P* < 0.01, compared with the 1 μM group.

To further confirm that SIK1 regulates the CRTC2 signaling pathway in HepG2 cells, we overexpressed SIK1 or inhibited its activity using the SIKs inhibitor HG-9-91-01, and determined the impacts on the expression levels of CRTC2 signaling-associated molecules. As shown in Figures 3A,B, SIK1 gene and protein expression levels displayed significant increases in the pGV230-SIK1 transfection group in comparison with the control groups at 48 h after transfection (*P* < 0.05). Meanwhile, compared with the control groups, the phosphorylation levels of CRTC2 at Ser171 were increased, while the protein levels of CRTC2, G6Pase, and PEPCK were all reduced upon pGV230-SIK1 transfection (*P* < 0.05) (Figure 3C). Then, immunocytochemistry demonstrated that SIK1 overexpression significantly reduced PEPCK protein amounts in HepG2 cells (Figure 3D), in agreement with immunoblotting results (Figure 3C).

In order to further verify the regulatory impact of SIK1 on the CRTC2 glucogenic signaling pathway, HepG2 cells after culture in normal glucose medium underwent treatment with the SIKs inhibitor HG-9-91-01. Interestingly, qRT-PCR results showed that SIK1 inhibition reduced CRTC2 mRNA amounts and increased G6Pase and PEPCK mRNA concentrations in a dose-dependent manner (*P* < 0.05) (Figure 3E). In addition, compared with the control groups, the HG-9-91-01 treatment groups exhibited significantly decreased protein amounts of total SIK1, phosphorylated SIK1, and phosphorylated CRTC2, as well as significantly increased protein amounts of total CRTC2, G6Pase, and PEPCK (*P* < 0.05), although the changes of CRTC2, G6Pase, and PEPCK protein expression levels did not seem to be overtly dose-dependent (Figure 3F).

### SIK1 Regulates the CRTC2 Signaling Pathway and Lipid Metabolism in Mouse Primary Hepatocytes

**Figure 4:**
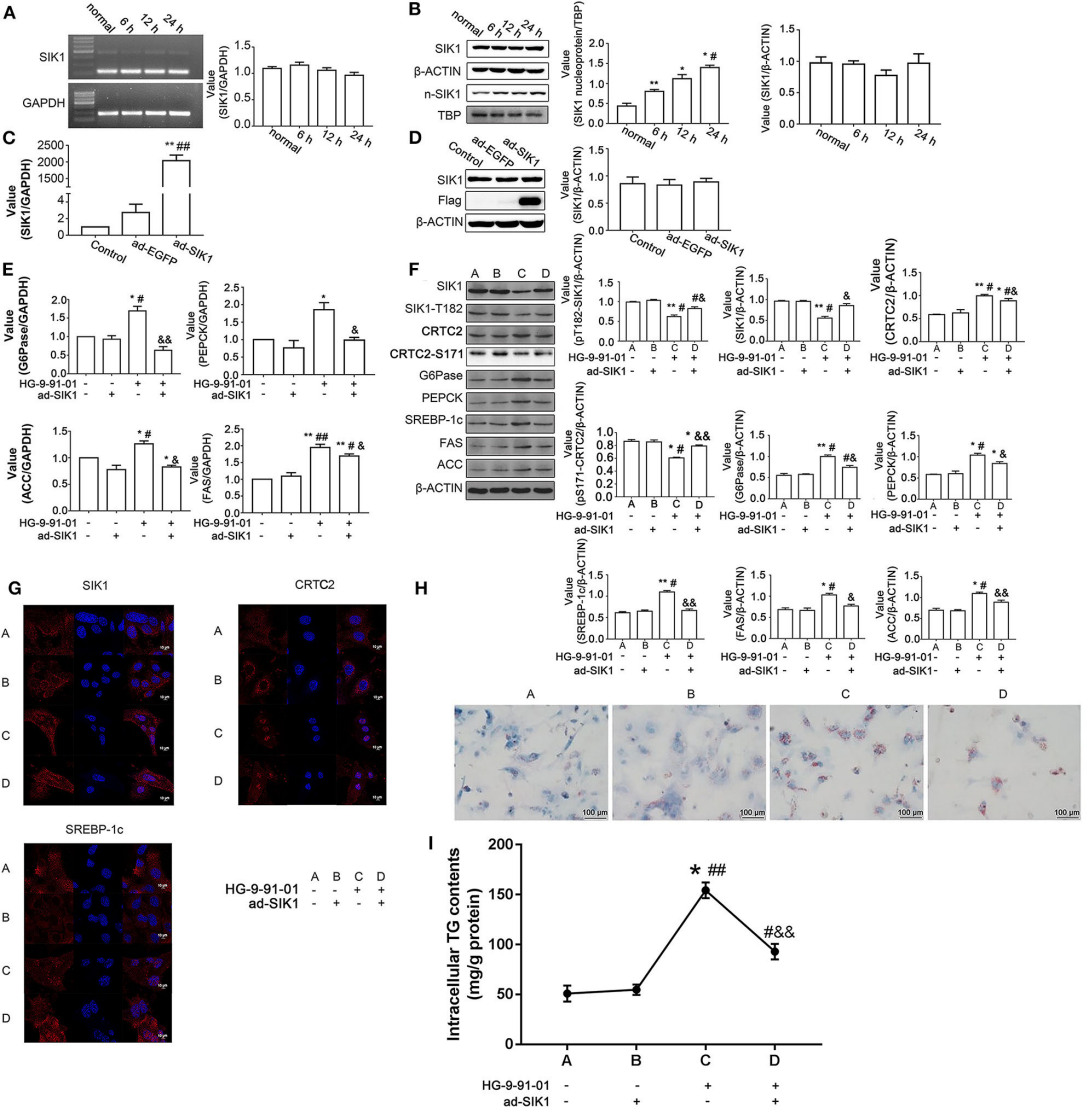
SIK1 regulates the CRTC2 signaling pathway and lipid metabolism in mouse primary hepatocytes. **(A,B)** Primary hepatocytes were isolated from wild type C57BL/6 mice and cultured in regular DMEM supplemented with a normal concentration of glucose (5.5 mmol/l D-glucose) or in DMEM supplemented with a high concentration of glucose (25 mmol/l D-glucose) for 6, 12, and 24 h. The mRNA levels of SIK1 **(A)** and the protein levels of total SIK1 in whole cells or nuclear extracts **(B)** were determined with PCR and immunoblotting, respectively. **(C,D)** Mouse primary hepatocytes were left untreated (Control), or infected with control adenovirus (ad-EGFP), or infected with Flag-tagged SIK1-expressing adenovirus (ad-SIK1), and cultured in regular medium for 48 h. The mRNA level of SIK1 **(C)** and the protein levels of SIK1/flag **(D)** were determined by qPCR and immunoblotting, respectively. **(E–H)** Mouse primary hepatocytes were left untreated, or treated with ad-SIK1 infection alone, or treated with 5 μM HG-9-91-01 incubation alone, or treated with simultaneous ad-SIK1 infection and 5 μM HG-9-91-01 incubation, and cultured in regular medium for 48 h. The mRNA **(E)** and protein **(F)** levels of the indicated molecules in mouse hepatocytes were determined by qPCR and immunoblotting, respectively. The cellular distributions of SIK1, CRTC2, and SREBP-1c in the indicated groups were evaluated by immunofluorescence **(G)**. Representative images showed the Oil Red O staining results of primary mouse liver cells after the indicated treatments **(H)**. **(I)** Quantitation of **(H)**. Scale bar, 10 μm in **(G)**, 100 μm in **(H)**. *n* = 3 for graphs in **(A–F)**. *P < 0.05, **P < 0.01, compared with the Control group; ^#^*P* < 0.05, ^##^*P* < 0.01, compared with the 6 h/ad-SIK1 group; ^&^*P* < 0.05, ^&&^*P* < 0.01, compared with the HG-9-91-01 alone group.

We isolated primary hepatocytes from wild type C57BL/6 mice, to examine whether the findings obtained in human cancerous HepG2 cells could be replicated in mouse primary hepatic cells. First, stimulation of mouse primary liver cells with high glucose medium did not result in significantly increased SIK1 mRNA (Figure 4A) and protein amounts (Figure 4B), but a time-dependent increase in nuclear expression of the SIK1 protein was identified (Figure 4B), which was consistent with the above data obtained in human HepG2 cells. We then established an adenovirus-mediated approach for the overexpression of Flag-tagged SIK1 in mouse hepatocytes and evaluated the impacts of ectopic SIK1 expression in HG-9-91-01-treated cells. Interestingly, ad-SIK1 adenovirus infection led to significantly increased SIK mRNA expression in mouse liver cells in comparison with un-infected and control virus-infected cells (Figure 4C) but resulted in largely unaltered protein expression of SIK1 (Figure 4D). While this treatment caused no significant changes in mRNA expression levels of CRTC2 signaling-associated molecules such as G6Pase and PEPCK, as well as lipid metabolism-related genes such as ACC and FAS, administration of HG-9-91-01 alone significantly elevated the mRNA amounts of these molecules (Figure 4E). Notably, additional infection of mouse hepatocytes with ad-SIK1 virus markedly reversed the effects of HG-9-91-01 treatment on the transcription of various molecules (Figure 4E). Similarly, immunoblotting also confirmed the same protein expression trends for these molecules (Figure 4F). In addition, immunofluorescence demonstrated that while single ad-SIK1 infection did not alter the cytoplasm-dominant distributions of SIK1, CRTC2, and SREBP-1c, treatment with HG-9-91-01 alone significantly promoted the nuclear import of these molecules, CRTC2 being the most evident example, and the addition of ad-SIK1 did not seem to modify the nuclear localization induced by HG (Figure 4G). Furthermore, Oil Red O staining (Figures 4H,I) also confirmed the trends of lipid metabolism changes upon SIK1 inhibition and ad-SIK1 infection in mouse primary liver cells, corroborating the qRT-PCR and immunoblotting data (Figures 4E,F).

## Discussion

Hepatic gluconeogenesis is essential in glucose homeostasis maintenance for meeting energy demands in both physiological and pathological conditions. The mechanisms underpinning transcriptional modulation and post-translational modifications (such as phosphorylation) during hepatic gluconeogenesis in response to high glucose are not fully understood. As shown above, the AMPK SIK1 was downregulated in human HepG2 cells and mouse primary hepatocytes under high glucose conditions, with reduced phosphorylation of SIK1 at the T182 site. High glucose may affect the synthesis or degradation of the total SIK1 protein, leading to changes in its phosphorylation activity on downstream CRTC2, rather than by regulating the phosphorylation level at T182, which will have to be confirmed. SIK1 was further shown to negatively regulate the CRTC2-mediated gluconeogenesis signaling pathway, as evidenced by upregulated CRTC2, G6Pase, and PEPCK upon SIK inhibition, and down-regulated CRTC2, G6Pase, and PEPCK upon SIK1 ectopic expression in liver cells. Therefore, SIK1 has an important function in high-glucose-dependent hepatic gluconeogenesis by regulating the CRTC2 signaling pathway.

CRTC2 has an essential function in regulating gluconeogenesis in hepatic cells as well as maintaining the stability of fasting blood glucose levels in normal conditions, and increased fasting blood glucose in diabetic patients has been shown to be associated with CRTC2 expression (3). CRTC2 functions as a co-activator of CREB, a member of the family of basic leucine zipper transcription factors. Upon phosphorylation, CREB interacts with CREB-binding protein and affects the expression of CRE downstream genes such as rate-limiting gluconeogenic enzyme genes (23, 24). Phosphorylation at Ser171 determines the transcriptional activity and intracellular localization of CRTC2. Dephosphorylation of CRTC2 at Ser171 promotes its entrance to the nucleus and binding to CREB, which further enhances CREB's transcriptional activity and upregulates downstream rate-limiting gluconeogenic enzyme genes, including G6Pase and PEPCK, to promote gluconeogenesis in the liver (23, 24). As shown above, high-glucose-induced SIK1 down-regulation was observed with significantly elevated expression of total CRTC2 in HepG2 cells, but their causal relationship is currently unknown. Nevertheless, phosphorylated CRTC2 at the S171 site was markedly reduced, which could explain the upregulated transcription of G6Pase and PEPCK. Consistently, Patel et al. showed that in primary rat hepatocytes, the SIKs inhibitor HG-9-91-01 increased intracellular CRTC2 dephosphorylation levels, upregulated rate-limiting gluconeogenic enzymes, and promotes hepatic gluconeogenesis (25). Moreover, our previous study suggested that high-glucose stimulation could reduce both protein and mRNA levels of SIK1 in rat HBZY-1 cells; in addition, SIK1 expression was also low in type 2 diabetic rat liver (17). Therefore, we hypothesized that SIK1 could modulate gluconeogenesis by altering the phosphorylation degree of CRTC2, and high glucose stimulation inhibits CRTC2 phosphorylation by SIK1. Nevertheless, the molecular mechanisms underlying the inhibitory effects of SIK1 on gene expression still need further experimental studies.

Immunofluorescence showed that high glucose exposure led to SIK1 enrichment in the nucleus of HepG2 cells. Meanwhile, the nuclear expression of SIK1 increased gradually with prolonged exposure to the high glucose environment. The nuclear export of SIK1 is closely related to its phosphorylation status and may significantly affect its activity (11). Ser577 is the autophosphorylation site of SIK1, which can be induced by PKA. Early studies showed that Ser577 autophosphorylation led to the nuclear export of SIK1 in mouse adrenocortical tumor cells, and mutation at this site resulted in SIK1 retention in the nucleus (9). In Cos-7 cells, Ser577 mutation of SIK1 significantly increases CRTC2 phosphorylation at Ser171 (11). The above findings suggest that Ser577 represents a critical factor affecting the intracellular distribution of SIK1. Thr182, another phosphorylation site of SIK1, is considered to contribute to CRTC2 phosphorylation by SIK1 (11). Therefore, Thr182 is the key to CRTC2 phosphorylation by SIK1. It was reported that the LKB1 introduction in HeLa cells results in autophosphorylation of SIK1 at the Ser577 site and causes a partial nuclear export of SIK1, while its mutation at Thr182 could eliminate the abovementioned export (11). It is inferred that phosphorylation of SIK1 at Thr182 may lead to autophosphorylation at the Ser577 site. In HepG2 cells, when phosphorylation of SIK1 at Thr182 was decreased, CRTC2 phosphorylation was also reduced. Therefore, the nuclear import of SIK1 is probably associated with the autophosphorylation of SIK1 at the Ser577 site.

This study demonstrated that SIK1 protein and mRNA levels in HepG2 cells in high-glucose environment gradually decreased, as well as the phosphorylation level at Thr182; meanwhile, SIK1 activity decreased and SIK1 tended to translocate into the nucleus. However, after high glucose stimulation in primary mouse hepatocytes, total SIK1 protein levels showed no significant change, which may be because SIK1 protein regulation by high glucose has species specificity. We further detected nuclear SIK1 protein levels, which were increased in primary mouse hepatocytes stimulated by high glucose. These findings indicated that in primary mouse hepatocytes, although the total protein expression of SIK1 was not downregulated by high glucose stimulation, this condition could promote its nuclear translocation.

The Thr812 site of SIK1 is closely related to its kinase activity (8, 26). We found that the phosphorylation level of Thr182 of SIK1 in HepG2 cells was decreased under high glucose conditions, accompanied by reduced total protein SIK1 levels (20). After treatment with HG-9-91-01 for 24 h, both HepG2 cells and primary mouse hepatocytes showed synchronously decreased total SIK1 protein amounts and Thr182 phosphorylation (Figures 3F, 4F). These data suggested that both high glucose conditions and HG-9-91-01 decreased SIK1 phosphorylation and activity. It was reported that HG-9-91-01 promotes the degradation of the SIK1 protein (27), suggesting that the decreased activity of SIK1 may be related to protein downregulation.

After HG-9-91-01 administration to primary liver cells from mice, we observed by immunofluorescence that SIK1 accumulated in the nucleus, and high sugar stimulation of HepG2 showed similar results. In both cases, SIK1 Thr182 site phosphorylation levels dropped, indicating that SIK1 translocation into the nucleus may be associated with decreased Thr182 phosphorylation. After LKB1 caused SIK1 phosphorylation at Thr182, the 14-3-3 protein binds to the phosphorylation site of SIK1 at Thr182, fixing it in the cytoplasm and enhancing its phosphorylation activity on CRTC2 in the cytoplasm (28). Thus, when phosphorylation at Thr182 is reduced, SIK1 is removed from the 14-3-3 protein and transferred from the cytoplasm to the nucleus. Furthermore, since phosphorylation at Thr182 is critical to SIK1 activity, it is associated with decreased SIK1 kinase activity (26). These findings suggest that decreased SIK1 activity results in intracellular transfer. Total SIK1 protein in primary mouse hepatocytes under high glucose did not change significantly compared with the normal glucose group, but the nuclear amounts of SIK1 were increased, suggesting that in high glucose stimulated primary mouse hepatocytes, SIK1 is translocated into the nucleus and its kinase activity decreases. This was consistent with the findings that high glucose stimulation inhibited SIK1 activity in HepG2 cells.

In order to further validate the regulatory effect of SIK1 on gluconeogenesis, SIK1 was overexpressed in HepG2 cells and mouse primary hepatocytes. As shown above, overexpression of SIK1 significantly promoted CRTC2 phosphorylation at the S171 site and downregulated CRTC2 at the protein level in the high glucose environment, thereby reducing the expression of rate-limiting gluconeogenic proteins in both the HepG2 cell line and primary liver cells. This suggests that overexpression of SIK1 can significantly inhibit gluconeogenesis induced by high glucose. In addition, the SIKs inhibitor HG-9-91-01 significantly reduced SIK1 protein amounts and markedly elevated the amounts of CRTC2 and rate-limiting gluconeogenic enzymes, suggesting that inhibiting SIK1 could promote gluconeogenesis. These conclusions were further supported by the reversal of HG-9-91-01-associated effects by adenovirus-mediated ectopic expression of SIK1 in mouse liver cells. In primary mouse hepatocytes, forced SIK1 expression not only promoted gluconeogenesis but also suppressed lipogenesis, as evidenced by altered SREBP-1c subcellular localization and reduced expression levels of SREBP-1c, FAS, and ACC upon additional ad-SIK1 infection, as well as diminished lipid accumulation indicated by Oil Red O staining. The above findings corroborated the data obtained in HepG2 cells, demonstrating that SIK1 expression is inversely correlated with the expression of lipogenic molecules and lipid biosynthesis (18).

Our data indicated that the expression of the SIK1 protein was upregulated after 48 h, which may be caused by the prolonged culture time, the consumption of glucose in the medium, and the inability to maintain a high-glucose environment. Because most glucose in the medium is used by 24 h, the time points used in the present study are 6, 12, and 24 h, and time points beyond 24 h were not used. In addition, the SIK1 expression patterns might be cell type- and species-specific (18, 29, 30), and additional studies are necessary to examine this issue. In addition, the nucleoproteins were measured in the present study, but the results about the cytoplasmic fraction were unsatisfactory and could not be analyzed in any reliable manner.

To sum up, the present study suggests that SIK1 has a key function in regulating gluconeogenesis in the liver, and should be considered a therapeutic target for regulating blood glucose. In a rat model of T2DM induced by a high-fat diet and low-dose STZ, the expression of SIK1 in the liver was decreased compared with the normal group, and the gluconeogenesis mediated by CRTC2 was enhanced. Moreover, the overexpression of SIK1 in the liver of the rats could downregulate the expression of gluconeogenic genes such as CRTC2, G6Pase, and PEPCK (20). Nevertheless, how high glucose stimulation downregulates SIK1, and the detailed mechanisms underlying SIK1-associated regulation of CRTC2 protein expression and phosphorylation still deserve further investigation.

## Conclusion

In conclusion, through examining the expression levels and phosphorylation status of relevant molecules upon exposure of human HepG2 cells or mouse primary hepatocytes in a high glucose environment, we demonstrated that SIK1 regulates the CRTC2 mediated gluconeogenesis signaling pathway in liver cells. Although more *in vivo* and clinical studies are required to further solidify our findings, the current study with *in vitro* experimental results still sheds new light on targeting SIK1 and the CRTC2 signaling for diabetes therapy.

## Data Availability Statement

The original contributions presented in the study are included in the article/supplementary material, further inquiries can be directed to the corresponding author/s.

## Ethics Statement

All experiments involving animals were approved by the University Committee on the Use and Care of Animals (UCUCA) of the Research Ethics Committee of Tongji Medical College, Huazhong University of Science and Technology.

## Author Contributions

CW substantially contributed to conception, acquisition and analysis of data, drafted the manuscript for important content, and critically revised the manuscript for important intellectual content. DS substantially contributed to design. JF contributed to the interpretation of data. XW substantially contributed to conception and critically revised the manuscript for important intellectual content. All authors gave final approval.

## Conflict of Interest

The authors declare that the research was conducted in the absence of any commercial or financial relationships that could be construed as a potential conflict of interest.

## Abbreviations

CRTC2: cAMP Regulated Transcriptional Co-activator 2
PEPC: phosphoenolpyruvate carboxykinase
SIK1: salt-inducible kinase 1
G6Pase: Glucose-6-Phosphatase
PEPCK: Phosphoenolpyruvate Carboxykinase
T2DM: type 2 diabetes mellitus
AMPK: AMP-activated protein kinase
ACTH: Adrenocorticotropic Hormone
LKB1: Liver Kinase B1
ALK5: activin receptor-like kinase 5
SREBP-1c: Sterol Regulatory Element-binding Protein-1c
FAS: Fatty acid synthase
ACC: Acetyl COA carboxylase
SPF: specific pathogen-free
UCUCA: University Committee on the Use and Care of Animals
Ad-EGFP: adenovirus containing enhanced green fluorescent protein
PFU: plaque forming units
qPCR: quantitative PCR
RIPA: radioimmunoprecipitation assay
SDS-PAGE: Sodium Dodecyl Sulfate-Polyacrylamide Gel Electrophoresis
TBST: Tris-buffered saline and Tween 20
ECL: enhanced chemiluminescence
FITC: Fluorescein isothiocyanate.
